# Inhibition of the TRIM24 bromodomain reactivates latent HIV-1

**DOI:** 10.1038/s41598-023-27765-3

**Published:** 2023-01-11

**Authors:** Riley M. Horvath, Zabrina L. Brumme, Ivan Sadowski

**Affiliations:** 1 Department of Biochemistry and Molecular Biology, Molecular Epigenetics Group, LSI, University of British Columbia, UBC, 2350 Health Sciences Mall, Vancouver, BC V6T 1Z3 Canada; 2 Faculty of Health Sciences, Simon Fraser University, Burnaby, BC Canada; 3 British Columbia Centre for Excellence in HIV/AIDS, Vancouver, BC Canada

**Keywords:** Biochemistry, Molecular biology, Virology

## Abstract

Expression of the HIV-1 genome by RNA Polymerase II is regulated at multiple steps, as are most cellular genes, including recruitment of general transcription factors and control of transcriptional elongation from the core promoter. We recently discovered that tripartite motif protein TRIM24 is recruited to the HIV-1 Long Terminal Repeat (LTR) by interaction with TFII-I and causes transcriptional elongation by stimulating association of PTEF-b/ CDK9. Because TRIM24 is required for stimulation of transcription from the HIV-1 LTR, we were surprised to find that IACS-9571, a specific inhibitor of the TRIM24 C-terminal bromodomain, induces HIV-1 provirus expression in otherwise untreated cells. IACS-9571 reactivates HIV-1 in T cell lines bearing multiple different provirus models of HIV-1 latency. Additionally, treatment with this TRIM24 bromodomain inhibitor encourages productive HIV-1 expression in newly infected cells and inhibits formation of immediate latent transcriptionally repressed provirus. IACS-9571 synergizes with PMA, ionomycin, TNF-α and PEP005 to activate HIV-1 expression. Furthermore, co-treatment of CD4 + T cells from individuals with HIV-1 on antiretroviral therapy (ART) with PEP005 and IACS-9571 caused robust provirus expression. Notably, IACS-9571 did not cause global activation of T cells; rather, it inhibited induction of IL2 and CD69 expression in human PBMCs and Jurkat T cells treated with PEP005 or PMA. These observations indicate the TRIM24 bromodomain inhibitor IACS-9571 represents a novel HIV-1 latency reversing agent (LRA), and unlike other compounds with this activity, causes partial suppression of T cell activation while inducing expression of latent provirus.

## Introduction

Despite intensive research over the past 40 years^1^, an estimated 38 million people are currently living with HIV-1, the vast majority of whom will require anti-retroviral therapy (ART) for the remainder of their lives^2^. Current ART does not target latently infected cells that harbor transcriptionally silenced provirus which represent a barrier for curing HIV-1 infection^3^, consequently various strategies to eliminate these cells are under intense investigation^4,5^. One potential strategy initially designated "Shock and Kill", would involve forcing reactivation of latent provirus such that latently infected cells become exposed to the host immune response and/ or additional therapeutic means for their elimination^6^. This potential strategy has prompted development of small molecule compounds capable of inducing expression of latent provirus, referred to as latency reversing agents (LRAs)^7^.

The 5' long terminal repeat (LTR) of integrated HIV-1 provirus contains numerous *cis*-elements for host cell factors that respond to signaling pathways involved in immune cell stimulation as well as growth factors and cytokines^8^. Latent provirus is established by multiple pathways in unstimulated cells, involving layers of regulation including down regulation of transcription factors responsive to immune signaling pathways and binding of transcriptional repressors to the 5' LTR that recruit histone deacetylases (HDACs) and histone methyltransferases (HMTs). HIV-1 expression in cells that revert to a resting state becomes shut down through epigenetic silencing and additional mechanisms that include loss of the viral transactivator protein Tat^10^. Additionally, ~ 50% of cells newly infected with HIV-1 harbor provirus that is transcriptionally repressed immediately^11^. This mode of latency is denoted early or immediate latency, and involves mechanism(s) that are influenced by signaling downstream of the T-cell receptor^12^, are capable of bypassing the function of Tat^13^, and is associated with binding of YY1 to the 5' LTR^14^.

Latent HIV-1 provirus in resting memory T helper cells becomes reactivated in response to T-cell receptor (TCR) engagement with antigen presenting dendritic cells. TCR signaling stimulates multiple transcriptional activators bound to the HIV-1 5ʹ LTR which cause recruitment of factors mediating transcription of the provirus genome^15^. Like many cellular genes, the 5ʹ LTR promoter of latent HIV-1 is associated with paused RNA Pol II^16,17^, and a key event for reactivation involves binding of viral Tat protein to nascent HIV-1 TAR RNA. Tat recruits the P-TEFb complex containing CDK9, which phosphorylates the pausing factors NELF and DSIF, and RNA Pol II CTD S2 to promote transcriptional elongation^18^. Reactivation of HIV-1 requires multiple factors specifically bound to the 5' LTR, including TFII-I which is constitutively associated with two *cis*-elements flanking the LTR enhancer region in combination with USF1/2^19,20^. TFII-I recruits the coactivator Tripartite-Motif containing protein 24 (TRIM24), which promotes transcriptional elongation from the LTR core promoter^21^, an effect that is associated with enhanced recruitment of CDK9 and increased RNA Pol II CTD S2 phosphorylation.

TRIM24 (TIF-1α) was discovered as a co-factor for nuclear hormone receptors^22^. TRIM24 contains conserved N-terminal RING, B-Box zinc finger, and coiled-coil domains, in addition to C-terminal PHD (plant homeodomain) and bromodomain motifs, which bind histone H3 with the combination of unmodified K4 and acetylated K23 epigenetic marks^23^. Overexpression of TRIM24 is associated with poor prognosis of a variety of cancers including breast, non-small cell lung, hepatocellular and glioblastomas^24,25^. Using structure guided design, a TRIM24 bromodomain binding compound, IACS-9571, was developed that inhibits interaction with histone H3K23ac peptides^26,27^. Interestingly, IACS-9571 was found to inhibit growth and metastatic invasive potential of glioblastomas^25^.

Here, we examine the effect of IACS-9571 on HIV-1 transcription, and surprisingly, find it causes reactivation of latent provirus and produces synergistic effects in combination with T cell signaling agonists. Treatment of T cells with IACS-9571 inhibits establishment of immediate latent provirus and encourages productive expression of HIV-1 in newly infected cells. IACS-9571 caused elevated association of TRIM24 with the LTR suggesting that inhibition of the bromodomain may promote redistribution of this factor from cellular genes. Treatment of CD4^+^ T cells from individuals with HIV-1 on ART with IACS-9571 in combination with Ingenol 3-angelate (PEP005), a small molecule protein kinase C (PKC) agonist^28^, caused robust viral transcription while inhibiting expression of T cell activation markers. These results indicate that the TRIM24 bromodomain inhibitor IACS-9571 represents a novel class of LRA which may prove useful for strategies to purge latently infected cells.

## Results

### IACS-9571 promotes HIV-1 expression

**Figure 1:**
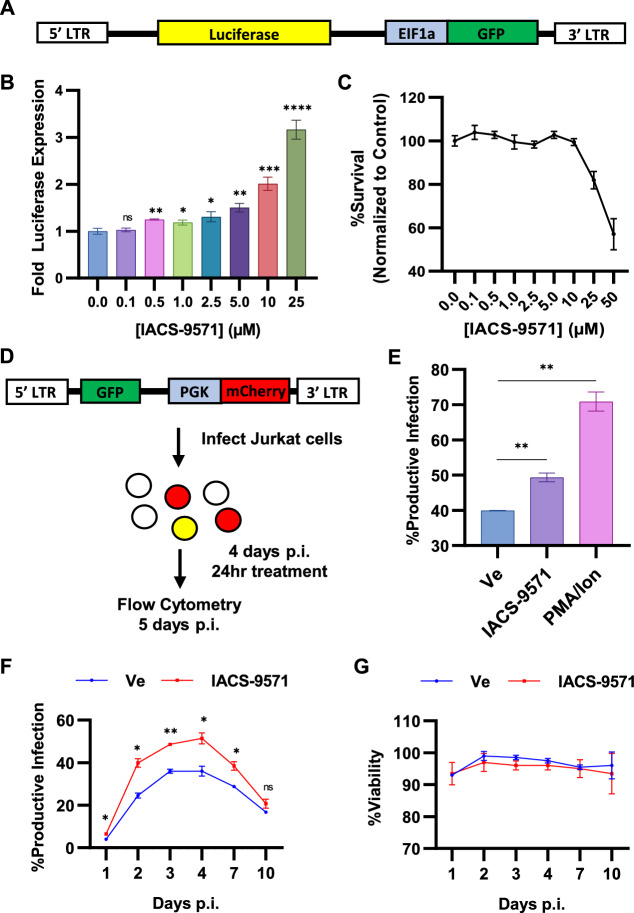
IACS-9571 promotes HIV-1 expression. (**A**) Schematic representation of HIV-1 mini-virus in the Jurkat Tat mHIV-Luciferase cell line, where luciferase is expressed from the 5' LTR as a fusion with Gag. (**B**) mHIV-Luciferase cells were treated with the indicated concentration of IACS-9571 for 4 h prior to luciferase assay. Results are an average of three determinations, and error bars represent standard deviation. (**C**) mHIV-Luciferase cell viability was determined after 4 h treatment with IACS-9571 at the indicated concentration. Viability assays were performed in duplicate with error bars representing standard deviation. (**D**) Depiction of Red-Green-HIV-1 (RGH) reactivation assays. RGH is a full-length replication incompetent HIV-1 virus with GFP expressed by the 5’ LTR as a fusion with Gag and *Nef* is replaced with a PGK promoter expressing mCherry^11^. Wildtype Jurkat T cells were infected with RGH at a low MOI; 4 days post-infection (p.i.), pools of cells were treated with 10 µM IACS-9571, 20 nM PMA/1 µM ionomycin, or left untreated for 24 h. Subsequently, productive and latently infected cells were measured by expression of GFP using flow cytometry. (**E**) Ratio of productively infected cells following RGH reactivation. Results are an average of two determinations, and error bars represent standard deviation. (**F**) Wildtype Jurkat T cells infected with RGH were treated with or without 10 µM IACS-9571 throughout the course of infection. Flow cytometry was performed on the indicated day post-infection (p.i.). Shown are the results of duplicate experiments, and error bars represent standard deviation. (**G**) Cell viability was determined at each time post-infection. Results of two measurements are shown, with error bars representing standard deviation.

We discovered that TRIM24 is recruited to the HIV-1 LTR by interaction with TFII-I, and this causes enhanced transcriptional elongation from the HIV-1 LTR^21^. Consequently, we examined what effect an inhibitor of the TRIM24 bromodomain might have on expression of HIV-1, using a Jurkat Tat mHIV-Luciferase cell line which bears an HIV-1 mini-virus where luciferase is expressed as a fusion with Gag (Fig. 1A)^14,21^. Surprisingly, IACS-9571 caused activation of luciferase expression in a dose-dependent manner, where treatment with 25 μM caused significant threefold induction in otherwise untreated cells (Fig. 1B), at concentrations that produce minimal toxicity (Fig. 1C). A similar effect of IACS-9571 was observed using JLat10.6 cells, which bear an HIV-1 provirus where *Nef* has been replaced by GFP (Fig. S1).

We also examined the effect of IACS-9571 on newly infected cells using a dual reporter Red-Green HIV (RGH) which enables detection of infected cells by expression of mCherry from an internal PGK promoter, and independent measurement of expression from the 5' LTR with a GFP reporter (Fig. 1D)^11^. Productively infected cells express both mCherry and GFP, whereas infected cells that produce immediate latent provirus only express mCherry (Fig. 1D). RGH infected cells treated with PMA and ionomycin 4 days post-infection produced ~ 70% productive infections, a significant increase from the ~ 40% productive infections in untreated (Ve) cells (Fig. 1E). IACS-9571 also caused an increase in the proportion of productively infected cells compared to the untreated control (Fig. 1E), indicating activation of latent provirus in a significant proportion of newly infected T cells. We examined the effect of IACS-9571 on virus expression over 10 days post infection where we found that ~ 35% of infected untreated cells (Ve) developed productive infections at 4 days post infection; the proportion of productively infected cells decayed from 4 to 10 days as more cells established latency (Fig. 1F)^13^. In contrast, cells treated with IACS-9571 generated a significantly greater proportion of productively infected cells, such that ~ 50% of infected cells expressed GFP 4 days post-infection (Fig. 1F, IACS-9571). Throughout the course of treatment, negligible effects upon cell viability were observed (Fig. 1G). These observations indicate that the TRIM24 bromodomain inhibitor dissuades establishment of immediate latency in newly infected cells and forces productive expression of viral RNAs from the 5' LTR.

### ***IACS-9571 stimulates HIV-1 expression in primary CD4***^+^***T cells ***ex vivo

**Figure 2:**
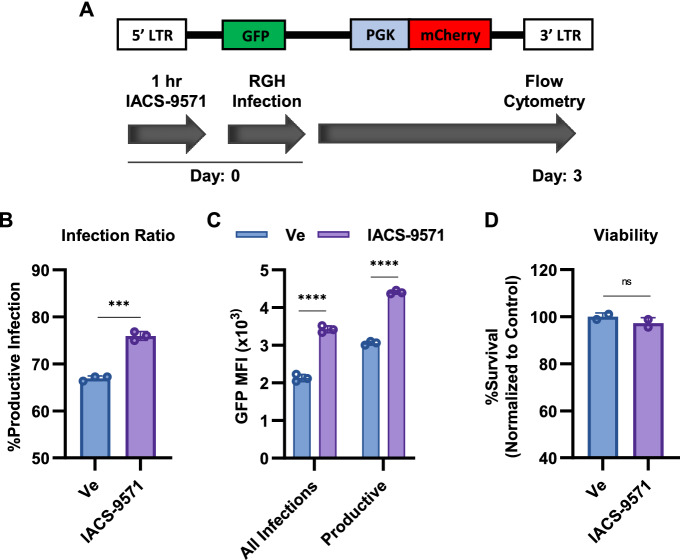
IACS-9571 stimulates HIV-1 expression in primary CD4^+^ T cells. (**A**) CD4^+^ T cells were pre-treated for one hour with vehicle control or 10 μM IACS-9571 and subsequently infected with RGH; flow cytometry was performed 3 days later. (**B**) The proportion of productively infected CD4^+^ T cells following 3 days treatment with 10 μM IACS-9571 or vehicle control is shown. Error bars represent standard deviation of 3 measurments. (**C**) As in Panel B but evaluating the Mean Fluorescence Intensity (MFI) of GFP expression. The GFP MFI was evaluated within the entire population of infected cells (All Infections) or within the population of cells that harbored productive infections (Productive). (**D**) Cells were treated as in (**A**,**B**) and viability was measured. Measurements were performed in duplicate with error bars representing standard deviation.

We also examined the effect of IACS-9571 on HIV-1 replication in primary CD4^+^ T cells. Here, we infected CD4^+^ T cells with RGH while treating with IACS-9571 or a vehicle control (Fig. 2A). Consistent with results using T cell lines, IACS-9571 caused an increase in the proportion of productive infections in normal CD4^+^ T cells (Fig. 2B,C). Interestingly, productive infections established during IACS-9571 treatment produced stronger expression of GFP from the 5ʹ HIV-1 LTR than provirus established in the absence of the drug (Fig. 2C, Productive). Notably, no change in CD4^+^ T cell viability was observed as a result of IACS-9571 treatment (Fig. 2D). These results confirm that IACS-9571 stimulates HIV-1 transcription in primary T cells.

### IACS-9571 produces synergistic effects with various LRAs

**Figure 3:**
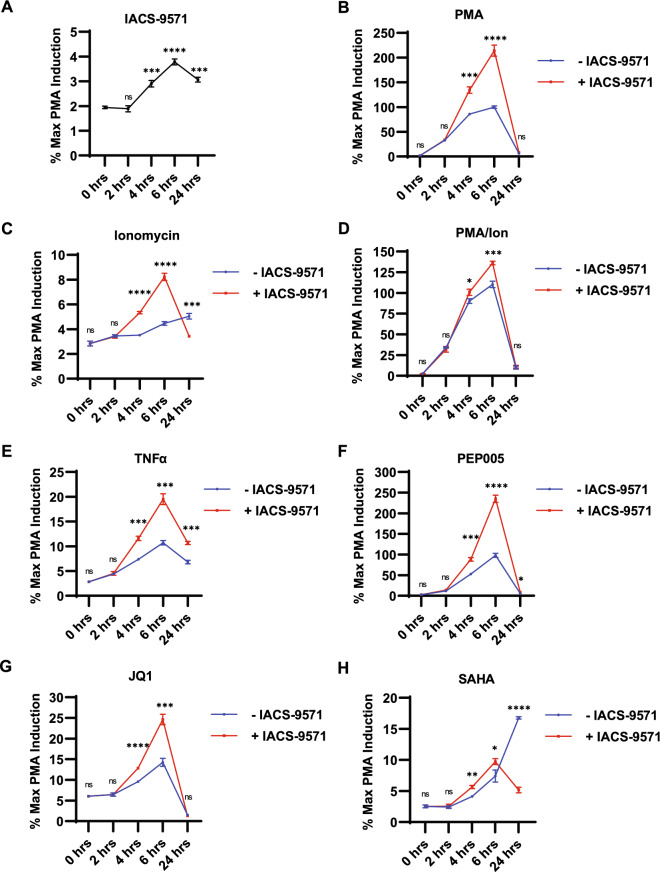
IACS-9571 causes synergistic activation T cell signaling agonists. (**A**–**H**) Jurkat Tat mHIV-Luciferase cells were treated with the indicated latency reversing agent, with or without 10 µM IACS-9571. Luciferase assays were performed at the indicated times. Results of three determinations are displayed, with error bars representing standard deviation. Concentration of agonists used were 20 nM PMA, 1 µM ionomycin, 10 ng/µL TNFα, 20 nM PEP005, 10 μM JQ1, 10 μM SAHA.

HIV-1 transcription is activated by multiple immune signaling pathways^8,15^, which control transcriptional activators bound to the LTR, including NF-κB, AP1, GABP/ Ets, NFAT, and USF1/2-TFII-I (RBF-2)^5,29^. We examined how IACS-9571 might influence responses to these signaling mechanisms by treatment in combination with signaling agonists. Jurkat mHIV-Luciferase cells (Fig. 1A) were treated with 10 µM IACS-9571, and luciferase expression was measured over the next 24 h, where we observed a modest, but significant ~ twofold induction of luciferase expression at 6 h (Fig. 3A). However, this concentration of IACS-9571 produced elevated HIV-1 expression in combination with any of the signaling agonists examined. This includes PMA (Fig. 3B,D,), which causes activation of multiple factors (NF-κB, AP1, GABP/ Ets); Ionomycin (Fig. 3C,D), which activates NFAT; TNF-α (Fig. 3E), which stimulates NF-κB and AP1; PEP005 (Fig. 3F), an LRA which acts through NF-κB; and JQ1, a BRD4 bromodomain inhibitor (Fig. 3G). Additionally, co-treatment with a combination of IACS-9571 and the HDAC inhibitor SAHA produced a modest increase in LTR-luciferase expression up to 6 h, an effect that is not only lost but ostensibly antagonized after 24 h (Fig. 3H). We examined the combined effect of IACS-9571 and the LRAs on stimulation of expression using Bliss independence modeling^30,31^, which indicated that IACS-9571 produced a synergistic effect with all of the T cell signaling agonists examined (Fig. S2). Notably, IACS-9571 and PEP005 induced the largest response of HIV-1 expression (Fig. 3F), and also produced the greatest degree of synergy amongst the LRAs examined (Fig. S2H). Co-treatment of IACS-9571 and SAHA produced a slight synergistic effect at 4 and 6 h with an antagonistic interaction emerging following 24 h (Fig. S2G). These observations are consistent with previous experiments demonstrating synergy between various LRAs that function through independent mechanisms^7^.

### TRIM24 is necessary for activation of HIV-1 expression, including by IACS-9571

**Figure 4:**
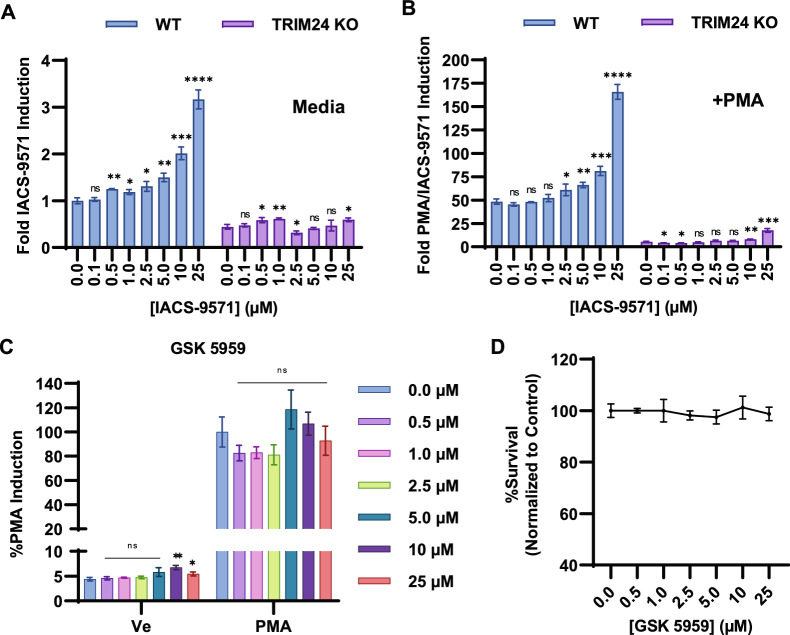
IACS-9571 requires TRIM24 for induction of HIV-1 expression. (**A**) Wildtype and *TRIM24* KO mHIV-Luciferase cells were treated with the indicated concentration of IACS-9571 for 4 h prior to luciferase assay. Assays were performed in triplicate, with error bars representing standard deviation. (**B**) Same as (**A**), but cells were co-treated with 20 nM PMA. Shown are the results of triplicate measurements, error bars represent standard deviation. (**C**) Jurkat mHIV-Luciferase cells were treated with the indicated concentration of GSK-5959 in the presence or absence of 20 nM PMA. Following 4 h ncubation, luciferase assay was performed. Shown are the results of three measurements, with error bars representing standard deviation. (**D**) Viability of mHIV-Luciferase cells were measured following 4 h incubation with the indicated concentration of GSK-5959. Assays were performed in duplicate with standard deviations displayed as error bars.

The TRIM24 bromodomain has similar structure to that of BRPF1, and IACS-9571 was shown to bind these bromodomains with similar affinity^27^. Consequently, it is possible that the effect of IACS-9571 on the HIV-1 LTR may be mediated by factor(s) other than TRIM24, including potentially BRPF1^27^. To examine this, we compared the effect of IACS-9571 on HIV-1 expression in the mHIV-Luciferase Jurkat cell line (Fig. 1A), and a derivative line bearing a CRISPR/ Cas9-mediated gene disruption of *TRIM24* (Fig. 4, TRIM24 KO)^21^. We observe dose-dependent induction in WT cells treated with IACS-9571, but this effect is completely inhibited in cells bearing the *TRIM24* KO (Fig. 4A). A similar effect was observed in cells treated with both IACS-9571 and PMA (Fig. 4B), where we observed a synergistic effect on HIV-1 expression in WT cells, but reactivation is significantly inhibited in the *TRIM24* KO line, with only slight activation at the highest concentration of IACS-9571. These results indicate that TRIM24 is the primary mechanistic target of IACS-9571 for induction of HIV-1 expression.

To further delineate effects of TRIM24 and BRPF1 bromodomain inhibition on HIV-1 expression, we treated mHIV-Luciferase Jurkat cells with GSK-5959, a potent and selective BRPF1 bromodomain inhibitor^32^. Unlike IACS-9571, we did not observe a dose-dependent effect on HIV-1 expression, however 10 μM GSK-5959 produced a modest but statistically significant 1.5-fold increase in HIV-1 expression in otherwise untreated cells (Fig. 4C, Ve). We also examined the effect of GSK-5959 in combination with PMA but found this co-treatment did not elevate HIV-1 expression relative to PMA alone (Fig. 4C, PMA). Of note, the concentrations of GSK-5959 applied did not affect cellular viability (Fig. 4D). We conclude that inhibition of the BRPF1 bromodomain by IACS-9571 does not contribute to reactivation of latent HIV-1. The small effect of IACS-9571 in combination with PMA in the *TRIM24* knockout cell line (Fig. 4B) may reflect residual off-target effects. All bromodomain inhibitors developed to date, including JQ1 (BRD4), GSK-5959 (BRPF1), and IACS-9571 (TRIM24/BRPF1) produce some off-target effects, and IACS-9571 and GSK-5959 share similar off-targets including BRPF2/ BRD1, BRPF3, BAZ2B, and TAF1^27,32^, although GSK-5959 does not bind the TRIM24 bromodomain^32^. Because GSK-5959 did not affect HIV-1 latency (Fig. 4C), it seems unlikely that the major effect of IACS-9571 on HIV-1 expression is produced by off target effects on additional bromodomain proteins.

### PROTAC-induced degradation of TRIM24 inhibits HIV-1 expression

**Figure 5:**
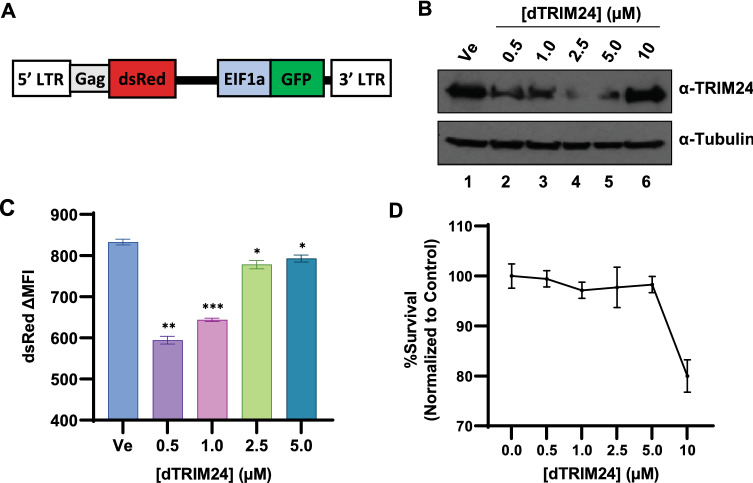
Effect of dTRIM24 on HIV-1 expression. (**A**) Schematic representation of HIV-1 mini-virus integrated in the Clone #11 Jurkat Tat cell line, where dsRed is expressed from the 5' LTR, while GFP is driven by the constitutive EIF1α promoter^13^. (**B**) Clone #11 cells were treated with the indicated concentration of dTRIM24 or left untreated (Ve) for 24 h. Immunoblots were performed on whole cell lysates, with antibodies against TRIM24 (top) or Tubulin (bottom). (**C**) Following 4 h pre-treatment with the indicated concentration of dTRIM24, Clone #11 cells were stimulated with 40 nM PMA for 20 h and analyzed by flow cytometry. Assays were performed in duplicate, with error bars representing standard deviation. (**D**) Cell viability was measured in cells as treated in (**C**). Results are an average of two determinations, and error bars represent standard deviation.

To examine the requirement of TRIM24 for induction of HIV-1 we used a Von Hippel-Lindau (VHL)-engaging functional degrader of TRIM24, designated dTRIM24^33^. This derivative is comprised of IACS-9571 conjugated to the VHL ligand VL-269 to produce a proteolysis-targeting chimeric compound (PROTAC), which promote degradation of target proteins by forcing interaction with the VHL ubiquitin ligase^34^. Treatment of Jurkat T cells bearing a mini-dual HIV-1 reporter provirus^13^ (Fig. 5A) with dTRIM24 causes degradation of TRIM24 at concentrations between 0.5 and 5 mM after 24 h, as determined by immunoblotting (Fig. 5B, lanes 2–5). As with previous observations, dTRIM24 mediated degradation is lost at higher concentrations (Fig. 5B, lane 6), an effect that may result from the molecule favoring binary interaction over ternary complex formation^33^. Having verified dTRIM24 concentrations that cause degradation, we measured induction of HIV-1 provirus in treated cells by analyzing dsRed expression (Fig. 5A) 20 h following stimulation with PMA. Consistent with our previous results^21^, we found that cells treated with concentrations of dTRIM24 that reduce TRIM24 protein levels also displayed a corresponding decrease in 5ʹ LTR dsRed expression (Fig. 5C), but without significantly affecting cell viability (Fig. 5D). This demonstrates that the TRIM24 bromodomain inhibitor IACS-9571 produces an opposite effect on HIV-1 transcription than does complete inhibition of TRIM24 protein function by dTRIM24 or knockout of the *TRIM24* gene^21^.

### The C-terminal TRIM24 bromodomain is dispensable for activation of HIV-1 expression

**Figure 6:**
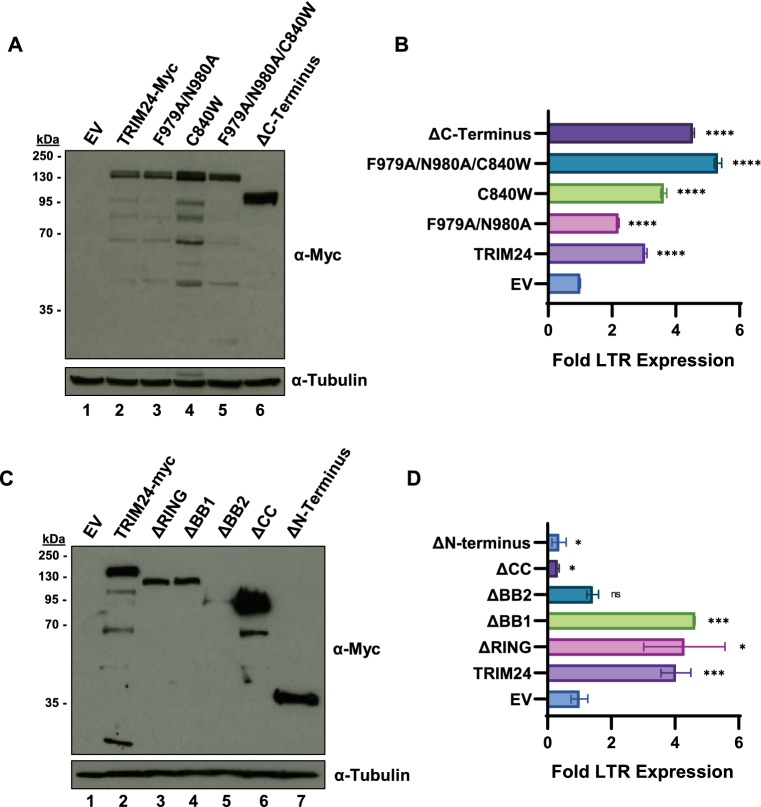
TRIM24 chromatin binding is dispensable for activation of HIV-1 transcription. (**A**,**C**) Two days post-transfection of HEK293T cells with the indicated construct, whole cell lysates were collected and immunoblotted using antibodies against Myc (top) or tubulin (bottom). (**B**,**D**) HEK293T cells were co-transfected with LTR-Luciferase reporter construct and the indicated TRIM24 WT or mutant expression vector. Two days post-transfection, LTR expression was analyzed by luciferase assay. Results are the average of three determinations with standard deviation indicated.

Because the IACS-9571 TRIM24 bromodomain inhibitor produces the opposite effect as *TRIM24* depletion, we examined whether the C-terminal bromodomain was necessary for activation of HIV-1 expression. For this, we co-transfected HEK293T cells with vectors expressing WT TRIM24, or mutants bearing ORF deletions and point mutations in the C-terminal PHD-bromodomain motifs (Fig. 6A,B*)* in combination with an HIV-1 LTR-luciferase reporter. Using this assay, we previously demonstrated that the effect of TRIM24 on HIV-1 expression does not require Tat^21^. Co-transfection of WT TRIM24 expression plasmid causes ~ threefold stimulation of HIV-1 luciferase expression (Fig. 6B, TRIM24). Interestingly, this effect does not require the C-terminal PHD-bromodomain region, as a deletion lacking the entire C-terminus (Fig. 6B, ∆C-Terminus) causes the same effect as WT TRIM24. Similarly, derivatives bearing amino acid substitutions in the C-terminal PHD-bromodomain motifs (F979A, N980A, C840W) that disrupt chromatin binding cause comparable levels of HIV-l expression as WT (Fig. 6B). In contrast, deletion of the complete N-terminus or the coiled-coil motif (Fig. 6C) prevented stimulation of HIV-1 expression (Fig. 6D). A mutant with deletion of the BB2 motif was less effective (Fig. 6D), but we note that this protein is expressed at significantly lower levels than the other TRIM24 derivatives (Fig. 6C). These results indicate that the bromodomain motif is not required for the effect of TRIM24 for reactivation of HIV-1 transcription.

### TRIM24 LTR occupancy is enhanced by IACS-9571

**Figure 7:**
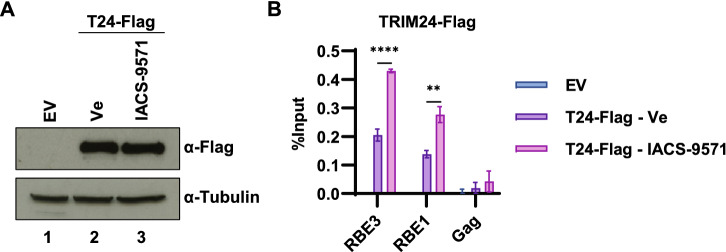
Association of TRIM24 with the HIV-1 LTR is enhanced upon IACS-9571 treatment. (**A**) Jurkat mHIV-Luciferase cells were transduced with lentivirus expressing Flag tagged TRIM24 (T24-Flag, Lanes 2, 3) or an empty vector (EV, Lane 1). TRIM24-Flag transduced cells were treated with 10 µM IACS-9571 (Lane 3) or left untreated (Ve, Lane 2). Whole cell lysates were immunoblotted using the indicated antibody. (**B**) TRIM24-Flag transduced cells were left untreated or incubated with 10 µM IACS-9571. Following 4 h, ChIP was performed using the indicated antibody. Results are the average of qPCR performed in triplicate with standard deviation shown.

We have previously shown that TRIM24 is associated with the HIV-1 LTR by interaction with TFII-I bound to the RBE3 and RBE1 *cis*-elements, positioned at -120 relative to the transcriptional start site, and immediately adjacent the core promoter, respectively^21^. The TRIM24 C-terminal tandem bromodomain—plant homeodomain (PHD) is binds histones with preference for H3K23ac and H3K4me0 marks, respectively^24^. Consequently, because TRIM24 is presumed to be predominantly recruited to chromatin via interactions mediated by the C-terminal bromo/ PHD domains, we examined the effect of bromodomain inhibition on recruitment of TRIM24 to the HIV-1 LTR. For this, we expressed Flag tagged TRIM24 in Jurkat cells harboring an HIV-1 reporter virus (Fig. 7A, lanes 2 and 3). Using ChIP-qPCR, we observed significantly enhanced interaction of TRIM24 with the LTR, at both RBE3 and RBE1 in cells treated with IACS-9571 (Fig. 7B). This observation is consistent with previous results indicating that TRIM24 is a limiting co-factor for activation of HIV-1 expression^21^, and suggests that inhibition of the TRIM24 bromodomain may inhibit global interaction with chromatin, thereby increasing the pool of TRIM24 available for recruitment by TFII-I at the HIV-1 LTR.

### IACS-9571 stimulates HIV-1 transcriptional elongation

**Figure 8:**
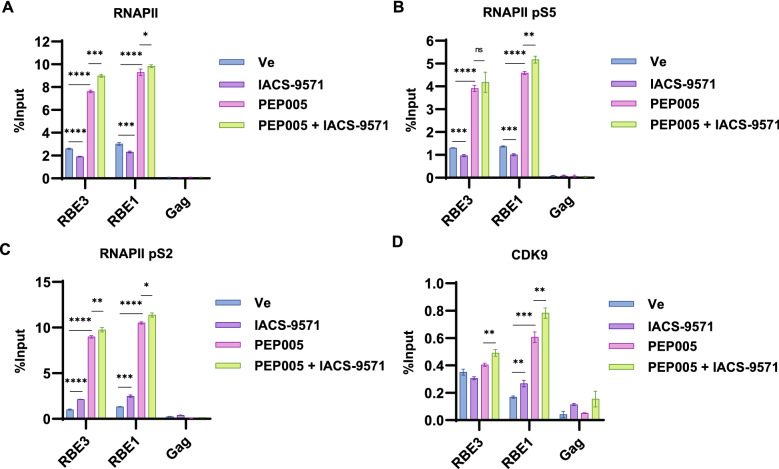
IACS-9571 stimulates association of transcriptional elongation factors with the HIV-1 LTR. (**A**–**D**) Jurkat mHIV-Luciferase cells were treated with 10 µM IACS-9571, 20 nM PEP005, 10 µM IACS-9571 and 20 nM PEP005, or DMSO (Ve). 4 h post-treatment ChIP was performed using the indicated antibody. Displayed are the average of replicate qPCR determinations with error bars representing standard deviation.

Our previous results indicate that TRIM24 causes enhanced elongation of transcription by RNA Polymerase II from the viral promoter^21^. Consequently, we examined whether treatment of cells with IACS-9571 also stimulates transcriptional elongation. We found that treatment of Jurkat mHIV-Luciferase cells (Fig. 1A) with IACS-9571 did not cause enhanced recruitment of RNAPII to the LTR, rather we observe a slight decrease in RNA PolII occupancy at the LTR in otherwise untreated cells (Fig. 8A, compare Ve vs IACS-9571). Initiation of transcription is associated with phosphorylation of RNAPII at the C-terminal domain (CTD) S5 by CDK7 of TFIIH^35^. As with total RNA Pol II occupancy, we observed slightly less pS5-modified RNAPII following IACS-9571 treatment as compared to untreated cells (Fig. 8B). Elongation of transcription from the LTR is stimulated by recruitment of P-TEFb, containing CDK9 which phosphorylates RNAPII CTD S2, among other factors, to promote transition from a paused to elongating transcription complex^36^. Interestingly, in contrast to RNAPII and CTD pS5, we found that treatment of mHIV-Luciferase cells with IACS-9571 caused elevated association of RNAPII pS2 with the LTR as compared to untreated cells (Fig. 8C). Additionally, IACS-9571 treatment caused significant enrichment of CDK9 (P-TEFb) at the HIV-1 core promoter (Fig. 8D, RBE1). These results are consistent with previous observations indicating that recruitment of TRIM24 to the HIV-1 LTR causes enhanced recruitment of P-TEFb/ CDK9, which promotes RNAPII CTD S2 phosphorylation and elongation of transcription^21^.

Results shown above (Fig. 3) indicate that IACS-9571 produces synergistic effects of HIV-1 expression in combination with T cell signaling agonists. We note that treatment with PEP005, an agonist of the PKC pathway^37^, causes significantly enhanced recruitment of RNAPII (Fig. 8A) and phosphorylation of CTD S5 (Fig. 8B) to the HIV-1 LTR, indicating that activation of factors regulated by T cell signaling have a significant effect on recruitment of RNA Pol II to the LTR promoter for promotion of transcriptional initiation. Co-treatment of cells with IACS-9571 and PEP005 causes corresponding elevated association of RNAPII, CTD pS5, CTD pS2, and CDK9 with the LTR (Fig. 8) which is consistent with the synergistic effect on HIV-1 reporter expression produced by the combination of these treatments.

### *IACS-9571 reactivates HIV-1 provirus in CD4* + *T-cells from individuals on ART*

**Figure 9:**
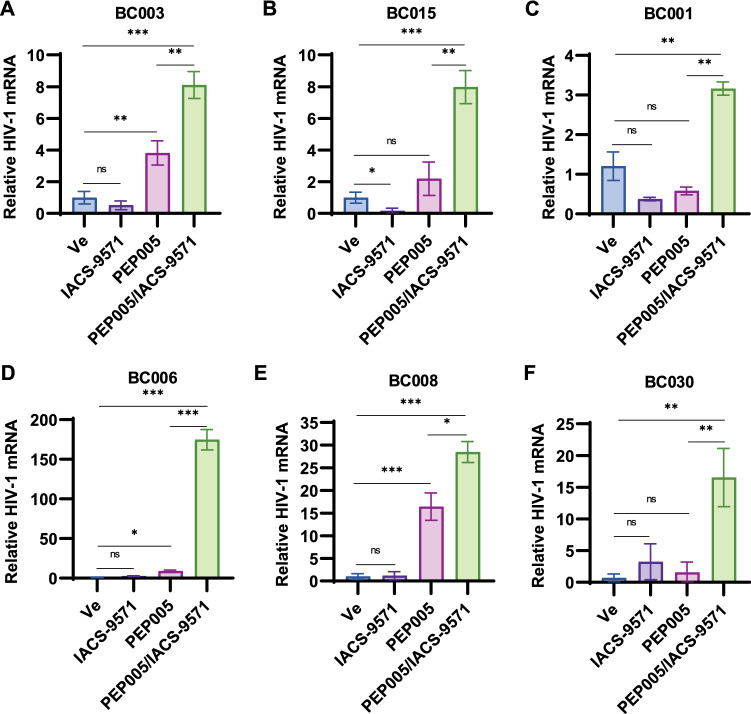
IACS-9571 activates HIV-1 expression synergistically with PEP005 in CD4^+^ cells from HIV-1 infected individuals. (**A**–**F**) CD4^+^ PBMCs isolated from HIV-1 patients on ART were treated with DMSO (Ve), 10 µM IACS-9571, 15 nM PEP005, or with 10 µM IACS-9571 and 15 nM PEP005 for 20 h. Following incubation, intracellular RNA was extracted and subjected to RT-PCR analysis with oligos targeting HIV-1 mRNA. The results of 2 or 3 RT-PCR determinations are displayed with standard deviation depicted as error bars.

To determine whether IACS-9571 was capable of affecting expression of HIV-1 in primary CD4^+^ lymphocytes, we examined the effect of treatment on CD4^+^ T-cells isolated from individuals with HIV-1 on ART. We found that treatment with 10 μM IACS-9571 on its own did not produce a detectable increase in HIV-1 mRNA in samples from 6 participants (Fig. 9, compare Ve and IACS-9571). This observation is not surprising, considering that CD4^+^ T cells containing integrated, replication competent provirus, are outnumbered by uninfected cells from individuals on ART by several orders of magnitude^5^. We examined the effect of the PKC agonist PEP005^7^ on HIV-1 expression in CD4^+^ T cells from these same donors, where we observed a significant increase in viral transcription in cells from two of the participants (Fig. 9A,E, BC003 and BC008), and a more modest effect on a third participant (Fig. 9D, BC006). Interestingly, with cells from all of the samples, co-treatment with IACS-9571 and PEP005 caused significant induction of HIV-1 mRNA (Fig. 9), a result that is consistent with the synergistic effects these agents have on HIV-1 expression in T cell lines (Fig. 3F, Fig. S2E).

### IACS-9571 partially suppresses T cell activation

**Figure 10:**
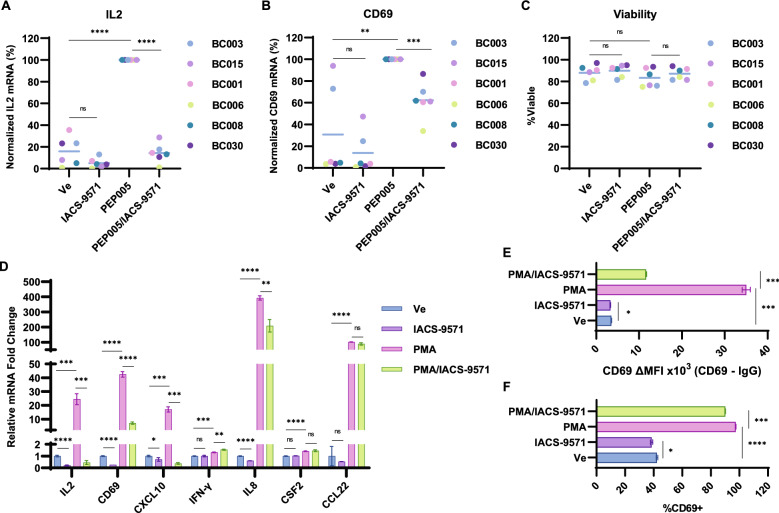
IACS-9571 inhibits a subset of T-cell activation response. (**A**,**B**) CD4^+^ cells from ART administered HIV-1 patients were treated with DMSO (Ve), 10 µM IACS-9571, 15 nM PEP005, or with 10 µM IACS-9571 and 15 nM PEP005 for 20 h. Following treatment, intracellular RNA was extracted and subject to RT-PCR using oligos that target IL2 (Panel A) or CD69 (**B**) mRNA. For each sample, RT-PCR was performed in replicate. (**C**) Viability of participant CD4^+^ PBMCs was assessed following treatment as in (**A**,**B**) using a Bio-Rad TC20 Automated Cell Counter. Results are an average of two independent measurements. (**D**) Jurkat mHIV-Luciferase cells were incubated with DMSO (Ve), 10 μM IACS-9571, 20 nM PMA, or a combination of 10 μM IACS-9571 and 20 nM PMA for 4 h. Following treatment, intracellular mRNA was isolated and RT-PCR was performed using oligos specific to the indicated transcript. Shown are the results of triplicate measurements with error bars representing standard deviation. (**E**,**F**) Jurkat mHIV-Luciferase cells were treated as in (**D**). CD69 was stained using PE-Cy7 conjugated antibody and flow cytometry was performed to measure Mean Fluorescent Intensity of CD69—PE-Cy7 (**E**) or the percent of cells positive for surface CD69 presentation (**F**). Assays were performed in duplicate with standard deviations represented by error bars.

An important consideration for development of LRAs as potential therapies is that treatment should produce robust and broad induction of HIV-1 expression without causing global T cell activation. To determine the potential effect of IACS-9571 on T cell activation, we examined expression of IL2 and CD69 mRNA in cells from participants using RT-PCR. Consistent with previous results^38^, treatment of CD4^+^ cells from participants with HIV-1 on ART with the PKC agonist PEP005 caused a significant increase in IL2 and CD69 mRNA (Fig. 10A,B, compare Ve vs PEP005). In contrast, treatment with IACS-9571 on its own did not cause elevation of either IL2 or CD69 transcripts. Moreover, we observed that treatment with IACS-9571 in combination with PEP005 resulted in robust suppression of IL2 induction while also generating a more modest ~ 40% reduction in CD69 mRNA (Fig. 10A,B, compare PEP005 vs PEP005/IACS-9571). Notably, the effect of IACS-9571 on HIV-1 latency reversal and T cell activation are independent of toxicity, as the treatments did not impact viability (Fig. 10C).

To further investigate the effect of IACS-9571 on T cell activation, we used the Jurkat T cell line to examine mRNA expression of T cell activation-induced genes. Similar to results with participant CD4^+^ samples, we found that IACS-9571 restricted IL2 and CD69 mRNA induction in response to T cell stimulation by PMA (Fig. 10D). Similarly, IACS-9571 inhibited PMA-induced expression of the cytokines CXCL10 and IL8 (Fig. 10D), but did not inhibit induction of IFN-γ, CSF2, or CCL22, indicating that this bromodomain inhibitor causes selective partial inhibition of T cell activation induced genes. We also examined the effect of IACS-9571 on expression of the CD69 T cell activation surface marker by flow cytometry (Fig. S4). Consistent with analysis of mRNA, PMA treatment caused accumulation of surface associated CD69 (Fig. 10E,F), but this effect was inhibited in cells treated with PMA in combination with IACS-9571 (Fig. 10E). Although surface associated CD69 was drastically reduced by IACS-9572 (Fig. 10E), the majority of cells still presented with CD69 (Fig. 10F), indicating that IACS-9571 causes a uniform decrease in CD69 expression rather than completely suppressing a limited segment of the cell population (Fig. S4C). Collectively, these results indicate that IACS-9571 causes reactivation of HIV-1 expression while partially suppressing T cell activation. This divergent functionality represents an important feature as no previous LRA was found to produce opposite effects for T cell activation and induction of HIV-1 expression^7^.

## Discussion

TRIM24 was initially identified as co-activator for various nuclear receptors^39^. This factor has E3 ubiquitin ligase activity, conferred by the N-terminal RING motif, and interacts with chromatin via the C-terminal PHD-bromodomain through binding of histone H3K4me0/H3K23ac^23^, but a mechanism for its function in transcriptional regulation had not been identified. We discovered that TRIM24 is recruited to the HIV-1 LTR by TFII-I, and promotes elongation of transcription through enhanced recruitment of P-TEFb/ CDK9 and phosphorylation of RNAPII CTD S2^21^. Consequently, we were surprised that the TRIM24 bromodomain inhibitor IACS-9571 caused elevated expression of HIV-1 provirus in otherwise untreated Jurkat T cells, an effect accompanied by enhanced association of TRIM24 with the promoter. Because TRIM24 seems to be a limiting factor for reactivation of HIV-1 transcription^21^, our observations suggest that IACS-9571 may inhibit interaction of this factor with global chromatin and consequently increases opportunity for recruitment to the LTR by TFII-I.

Global effects on transciption by IACS-9571 are largely divergent from that caused by dTRIM24 facilitated protein degradation^33^. In this study, we discovered HIV-1 expression is differentially affected by IACS-9571 and dTRIM24 treatment. IACS-9571 causes reactivation of HIV-1 provirus at similar concentrations, and as effectively, as most previously characterized LRAs^7^. Conversely, we found that forced degradation of TRIM24 with dTRIM24^27,33^ inhibited HIV-1 expression at concentrations where it caused reduction of TRIM24 protein levels. This latter observation, in combination with our previous results indicating that *TRIM24* KO inhibits HIV-1 reactivation^21^ suggests that full inhibition of TRIM24 function, or at least interaction with TFII-I on the LTR, represent prospective latency promoting targets. Overall, these observations indicate that TRIM24 is an important potential target than can be modulated to enhance or repress HIV-1 provirus expression for therapeutic strategies.

TRIM24 contributes to both positive and negative effects on gene expression. In RNA-seq analysis of *TRIM24* KO cells we observed differential effects on nearly 2000 genes compared to WT, with approximate equal representation of up and down regulated genes^21^. Details regarding mechanisms for the repressive effect of TRIM24 is limited, but was reported to involve inhibition of transcriptional activation by the retinoic acid receptor^40^ and Smad4^41^ by ubiquitylation of these factors. These observations suggest that TRIM24 mediated ubiquitylation may inhibit one or more factor(s) bound to the 5' LTR to mediate viral expression. In this view, it is possible that TRIM24 function for repression or activation of HIV-1 expression is altered by T cell signaling mechanisms. TRIM24 is recruited to the LTR by TFII-I^21^, a protein that functions for both repression and activation of HIV-1 provirus expression^19,29,37^. Consequently, it is possible that the divergent effects of factors bound to the conserved RBE3 and RBE1 elements may be mediated by differentially regulated functions of TRIM24 and that these functions are altered by IACS-9571. Although the TRIM24 RING domain was found to be dispensable for activation of LTR transcription in HEK293T cells (Fig. 6C,D), further work will be required to examine the potential effects of TRIM24 mediated ubiquitylation in T cell lineages.

Alternatively, the latency reversing effect of IACS-9571 may be produced by inhibition of its interaction with H3K4me0/H3K23ac modified chromatin on cellular genes. This effect might provide higher levels of TRIM24 available for recruitment to the LTR. Consistent with this view we observe enhanced levels of TRIM24 associated with the LTR in cells treated with IACS-9571 (Fig. 7). Furthermore, we find that TRIM24 mutant proteins bearing deletion of the C-terminal region spanning the histone binding motifs, or point mutations within those motifs, do not affect the capability of this factor to stimulate HIV-1 transcription (Fig. 6A,B). These observations incidentally suggest that the histone binding function of TRIM24 is not directly required for its effect on stimulating transcriptional elongation from the HIV-1 LTR. However, a more detailed understanding of TRIM24 function will be required to elucidate the mechanism for IACS-9571 as an LRA.

CD4^+^ T-helper 2 cells lacking TRIM24 display dampened inflammation response characterized by reduced expression of many cytokines and chemokines^42^. Here, we found that unlike most other LRAs, IACS-9571 inhibits expression of genes associated with T cell activation in CD4^+^ cells from individuals with HIV-1 on ART, and in Jurkat T cells stimulated with PMA. An important consideration for LRA development is their effectiveness for reactivation of HIV-1 provirus without causing global T cell activation^7^. Unchecked activation of T cells can produce a response known as cytokine storm, typified by excess production of proinflammatory cytokines which can cause acute respiratory distress syndrome. Cytokine storm caused by hyperactivation of T cells contributes significantly to pathology of COVID-19^43^ and influenza^44^. Our observation that IACS-9571 inhibits genes associated with T cell activation, but causes activation of HIV-1 illustrates a unique capability of this novel LRA. The differential effect of TRIM24 for regulation of HIV-1 and T cell response must be mediated by differential requirement for the C-terminal bromodomain. A more detailed understanding of the mechanistic significance of histone H3K23 acetylation will be required to elucidate the effect of IACS-9571 on HIV-1 transcription. Nevertheless, considering the unique effects this compound has on HIV-1 provirus and T cell activation we suggest that IACS-9571, and its derivatives, may prove useful for strategies involving LRAs to purge latently infected cells from HIV-1 infected individuals. IACS-9571 causes cause synergistic reactivation of HIV-1 expression in combination with PEP005 (Fig. 9), and we suggest this combination may prove useful for potential therapies involving reactivation of virus expression. Accordingly, we note this compound was proposed as therapy for glioblastomas where TRIM24 is a contributing factor for malignancy^45^, and that PEP005 is presently in clinical trials for skin cancers^46^.

## Materials and methods

### Cell and virus culture

Jurkat and HEK293T cells were maintained as previously described^20^. VSV-G pseudotyped viruses were produced by co-transfection of HEK293T cells with psPAX and pHEF-VSVg as previously described^20^. Human Peripheral Blood CD4^+^ T cells were purchased from STEMCELL Technologies (Catalog # 200-0165). T cells were cultured in RPMI supplemented with 10% FBS, penicillin (100 U/mL), streptomycin (100 mg/mL), and 30 U/mL IL2, and infection with RGH was performed as previously described^47^. Briefly, cells were first incubated for three days with Dynabeads™ Human T-Activator CD3/CD28 beads. The beads were removed and cells were infected with RGH at a multiplicity of infection (M.O.I.) that resulted in less than 8% of the population infected.

Peripheral Blood Mononuclear Cells (PBMC) from participants with HIV-1 on ART were isolated from whole blood by density gradient centrifugation using Lymphoprep™ and SepMate™ tubes (StemCell Technologies), and cryopreserved^48^. Upon thawing, PBMCs were cultured in RPMI supplemented with FBS (10%), penicillin (100 U/mL), and streptomycin (100 mg/mL). Samples from donor participants were collected with informed consent obtained from all subjects and/or their legal guardian, and handled using methods in accordance with guidelines and protocols approved by the University of British Columbia Clinical Research Ethics Board, certificate H16-02474.

### Immunoblotting

Western blotting was performed as previously described^48^. Antibodies were as follows: Tubulin, Abcam ab7291; Flag, Sigma Aldrich F3165; Myc, Santa Cruz sc-40; TRIM24, Proteintech 14208-1-AP; TFII-I, Abcam ab134133; Goat Anti-Rabbit-HRP, Abcam ab6721; Goat Anti-Mouse-HRP, Pierce 1858413.

### Chromatin immunoprecipitation

Jurkat mHIV-Luciferase cells were fixed with 1% formaldehyde (Sigma-Aldrich) for 10 min at room temperature (3 × 10^7^ cells/IP). 125 mM glycine was added for 5 min to quench cross-linking, and cells were subsequently washed with PBS on ice. Cells were lysed in NP-40 Lysis Buffer (0.5% NP-40, 10 mM Tris–HCl pH = 7.8, 3 mM MgCl_2_, 1 × PIC, 2.5 mM PMSF) for 15 min on ice. Following sedimentation, nuclei were resuspended in sonication Buffer (10 mM Tris–HCl pH = 7.8, 10 mM EDTA, 0.5% SDS, 1 × PIC, 2.5 mM PMSF) and sonicated using a Covaris S220 Focused-ultrasonicator to produce sheared DNA (2000–200 bp). The soluble chromatin fraction was collected and snap frozen in liquid nitrogen. Chromatin concentrations were normalized among samples and pre-cleared with Protein A/G agarose (Millipore, 100 μL/IP). The chromatin samples were split in two and diluted with IP buffer (10 mM Tris–HCl pH = 8.0, 1.0% triton X-100, 0.1% deoxycholate, 0.1% SDS, 90 mM NaCl, 2 mM EDTA, 1 × PIC); samples were immunoprecipitated with the indicated specific antibody or control IgG. Antibodies for ChIP were: TRIM24, Proteintech 14208-1-AP; RNAPII, Abcam ab26721; RNAPII pS5, Abcam ab5408; RNAPII pS2, Abcam ab238146; CDK9, Abcam ab239364; Flag, Sigma Aldrich F3165; Mouse IgG, Santa Cruz sc-2025; Rabbit IgG, Abcam ab1722730. The chromatin/ antibody mixtures were incubated 1 h at 4 °C with rotation. Pre-washed Protein A/G agarose beads (40 μL/ IP) were then added and the samples were incubated overnight at 4 °C with rotation. Bead complexes were washed 3 × in Low Salt Wash Buffer (20 mM Tris–HCl pH = 8.0, 0.1% SDS, 1.0% Triton X-100, 2 mM EDTA, 150 mM NaCl, 1 × PIC) and 1 × with High Salt Wash Buffer (same but with 500 mM NaCl). Elution and crosslink reversal was performed by incubating 4 h at 65 °C in elution buffer (100 mM NaHCO_3_, 1% SDS) supplemented with RNase A. DNA was purified using the QIAQuick PCR purification kit (QIAGEN) and ChIP DNA was analyzed using the Quant Studio 3 Real-Time PCR system (Applied Biosystems). The percent input value of the IgG sample was subtracted from the specific antibody percent input value of the corresponding sample. Oligos used for ChIP-qPCR were: RBE3, Fwd 5ʹ AGCCGCCTAGCATTTCATC, Rev 5ʹ CAGCGGAAAGTCCCTTGTAG; RBE1, Fwd 5ʹ AGTGGCGAGCCCTCAGAT, Rev 5ʹ AGAGCTCCCAGGCTCAAATC; Gag, Fwd 5ʹ AGCAGCCATGCAAATGTTA, Rev 5ʹ AGAGAACCAAGGGGAAGTGA.

### Luciferase reporter assays

Luciferase expression assays from transiently transfected cells were performed as previously described^21^. Polyethylenimine (PEI) transfection of HEK293T cells were performed in 96-well plates seeded with 2 × 10^4^ cells per well one day prior to transfection. Cells were co-transfected with 10 ng of pGL3 LTR reporter plasmid and 100 ng expression vector; luciferase activity was measured 24 h post-transfection. For Jurkat luciferase reporter assays, 96-well plates were seeded with 1 × 10^5^ cells in 100 µL media, and luciferase activity was measured after the indicated time of treatment. Measurements were performed using Superlight™ luciferase reporter Gene Assay Kit (BioAssay Systems) as per the manufacturer’s instructions, and activity was determined by a VictorTM X3 Multilabel Plate Reader.

### Q-*RT-PCR*

Following the indicated treatment, RNA was extracted from cells using an RNeasy Kit (Qiagen) and subsequently analyzed with the Quant Studio 3 Real-Time PCR system (Applied Biosystems) using *Power* SYBR® Green RNA-to-CT™ 1-Step Kit (Thermo Fisher) as per the manufacturer’s instructions. Primers are as follows: IL2, Fwd 5ʹ AACTCACCAGGATGCTCACA, Rev 5ʹ GCACTTCCTCCAGAGGTTTGA; CD69, Fwd 5ʹ TCTTTGCATCCGGAGAGTGGA, Rev 5ʹ ATTACAGCACACAGGACAGGA; CXCL10, Fwd 5ʹ AAGTGGCATTCAAGGAGTACCT, Rev 5ʹ GGACAAAATTGGCTTGCAGGA; IFN-γ, Fwd 5ʹ ATTCGGTAACTGACTTGAATGTCC, Rev 5ʹ CTCTTCGACCTCGAAACAGC; IL8, Fwd 5ʹ ACTGAGAGTGATTGAGAGTGGAC, Rev 5ʹ AACCCTCTGCACCCAGTTTTC; CSF2, Fwd 5ʹ ACCTGCCTACAGACCCGCCT, Rev 5ʹ GAAGTTTCCGGGGTTGGAGGGC; CCL22, Fwd 5ʹ CGCGTGGTGAAACACTTCTAC, Rev 5ʹ GCCACGGTCATCAGAGTAGG; HIV-1 mRNA, Fwd 5ʹ CTTAGGCATCTCCTATGGCAGGA, Rev 5ʹ GGATCTGTCTCTGTCTCTCTCTCCACC.

### Flow cytometry

Cells were treated as indicated in the legends. Subsequent to treatment, cells were collected, resuspended in PBS, and analyzed on a BD Biosciences LSRII-561 system where threshold forward scatter (FSC) and side scatter (SSC) parameters were set so that only a homogenous population of live cells were counted (Fig. S4A). Data was analyzed and mean fluorescent intensities were determined using FlowJo software (TreeStar). For surface staining of CD69, 1 × 10^6^ mHIV-Luciferase cells were treated for 4 h in the presence of DMSO (Ve control), 10 μM IACS-9571, 10 nM PMA, or 10 nM PMA and 10 μM IACS-9571. Following treatment, blocking was performed by adding CD16/CD32 Rat anti-Mouse, Clone: 2.4G2 (BD Biosciences 553141) and incubating on ice for 15 min. Subsequently, PE-Cy™7 Mouse IgG1 κ Isotype Control (BD Biosciences 557872) or PE-Cy™7 Mouse Anti-Human CD69 (BD Biosciences 561928) was added, and the samples were incubated 30 min on ice. Samples were then washed 2 × with ice cold PBS and resuspended in PBS for analysis on the BD Biosciences LSRII-561 system as stated.

### Quantitative analysis of IACS-9571 and latency reversing agent interaction

Bliss independence modeling was used as previously described^30,31^ to statistically assess the activity of IACS-9571 in combination with various LRAs on HIV-1 expression. The equation *Fa*_xy, P_ = *Fa*_*x*_ + *Fa*_*y*_ – (*Fa*_*x*_)(*Fa*_*y*_) defines the Bliss independence model, in which *Fa*_xy, P_ is the predicted fraction affected by a combination of drug *x* and drug *y* that is derived from the experimentally observed fraction affected by drug *x* (*Fa*_*x*_) and drug *y* (*Fa*_*y*_) individually. Comparison of the predicted combinatorial affect (*Fa*_xy, P_) with the experimentally observed impact (*Fa* _*xy*,*O*_) was then performed: ∆*Fa* _*xy*_ = *Fa* _*xy*,*O*_ − *Fa* _*xy*,*P*_. If ∆*Fa* _*xy*_ is greater than 0, the combination of drugs *x* and *y* exceed that of the predicted affect indicating that the drugs display synergistic interaction. If ∆*Fa* _*xy*_ = 0, the drug combination follows the Bliss model for independent action. If ∆*Fa* _*xy*_ is less than 0, the drug interaction is antagonistic as the observed effect of the drug combination is less than predicted. In this analysis, the fraction affected was calculated as follows: *Fa*_*x*_ = (HIV expression of drug *x* at the indicated time – HIV expression at time 0 h) / (Max HIV expression observed over the time course – HIV expression at time 0 h).

### Statistical analyses

Details of statistical analysis are indicated in figure legends. Mean is shown with standard deviations. Unpaired sample *t*-tests were performed using GraphPad Prism 9.0.0, and statistical significance is indicated at *P < 0.05, **P < 0.01, ***P < 0.001, or ****P < 0.0001.

## Supplementary Information

Supplementary Information.

## Data Availability

All data pertaining to the findings of this study are available within the article and its Supplementary Information, or are available from the corresponding author, I. Sadowski, ijs,ubc@gmail.com.
