# Supplementary material for: Inhibition of the TRIM24 bromodomain reactivates latent HIV-1
Source: Sci Rep. 2023 Jan 11;13:556. doi: 10.1038/s41598-023-27765-3 (PMC9832417; doi:10.1038/s41598-023-27765-3)
Supplement: Supplementary file 1 — Supplementary Information. [file 41598_2023_27765_MOESM1_ESM.pdf]

Figure S1. IACS-9571 promotes HIV-1 expression. Panel A: Schematic representation of HIV-1 virus integrated in the JLat10.6 cell line. The full-length virus expresses GFP in place of *Nef* with mutations to *Env* rendering it replication incompetent. **Panel B:** JLat10.6 cells were treated with IACS-9571 at the indicated concentration for 24 hrs and subsequently analyzed by flow cytometry. Results are the average of two measurements, error bars represent standard deviation. **Panel C:** JLat10.6 cell viability was determined following 24 hrs treatment with the indicated concentration of IACS-9571. Assays were performed in duplicate and error bars depict standard deviation.

Figure S2. Quantitative analysis of synergy between IACS-9571 and latency reversal agents. Panels A – G: Calculation of synergy between IACS-9571 and the indicated latency reversal agent at the given time point was performed using Bliss Independence Modelling. Data are presented as the difference between the predicted and the observed fractional HIV-1 expression response to the given drug combination. See Materials and Methods for more details. **Panel H:** As in Panels A – G but calculated at 6 hrs post-incubation with the combination of IACS-9571 and the indicated compound.

Figure S3. Jurkat Tat Clone #11 is responsive to IACS-9571. Panel A: Clone #11 cells were treated with 40 nM PMA or 40 nM PMA and 10 μM IACS-9571. Flow cytometry was performed following 24 hrs incubation. Displayed are the results of duplicate experiments, with error bars representing standard deviation. **Panel B:** Viability of Clone #11 cells treated with 40 nM PMA and the indicated concentration of IACS-9571 was determined following 24 hrs

incubation. Displayed are the results of two determinations with error bars depicting standard deviation.

Figure S4. Analysis of surface CD69 by flow cytometry. Panel A: Representative scatter plots depicting the gating strategy employed. Threshold forward scatter (FSC) and side scatter (SSC) settings were set as to isolate a homogeneous population of live cells that were then gated (Left, Lymphocytes). The gated population was then examined for fluorescence of PE-Cy7 (Right). **Panel B, C:** Representative flow cytometry scatter plots detecting surface associated CD69. Jurkat mHIV-Luciferase cells were treated with a DMSO control (Ve), 10 μM IACS-9571, 20 nM PMA, or 10 μM IACS-9571 in combination with 20 nM PMA. Following treatment, cells were stained with PE-Cy7 conjugated antibodies against IgG (Panel B) or CD69 (Panel C) and analyzed by flow cytometry.

Figure S5. Full length western blots. Protein extract samples were analyzed by SDS-PAGE and blotted to nitrocellulose membranes. Membranes were probed with the indicated antibody and detected with enhanced chemiluminescence (ECL) and exposure to Hyperfilm™ ECL™ film. Shown are full uncropped images of the developed films.

Figure S1

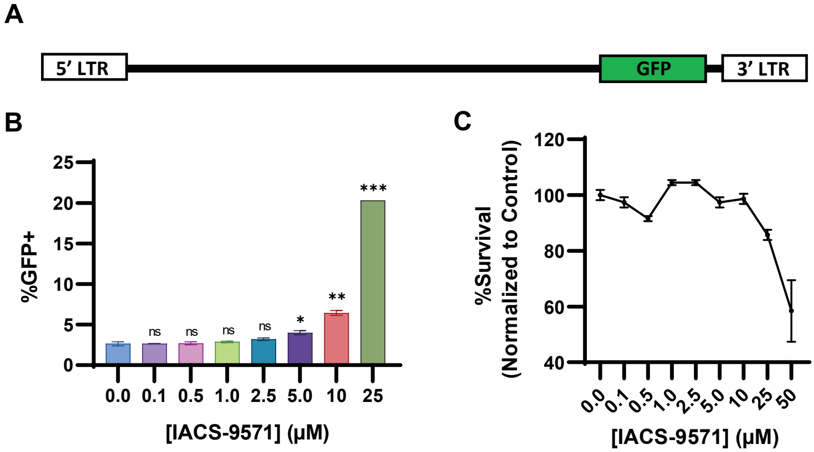
A
5' LTR
GFP
3' LTR
B
%GFP+
25
20
15
10
5
0
ns
ns
ns
ns
*
**
***
0.0
0.1
0.5
1.0
2.5
5.0
10
25
[IACS-9571] (μM)
C
%Survival
(Normalized to Control)
120
100
80
60
40
0.0
0.1
0.5
1.0
2.5
5.0
10
25
50
[IACS-9571] (μM)

Figure S2

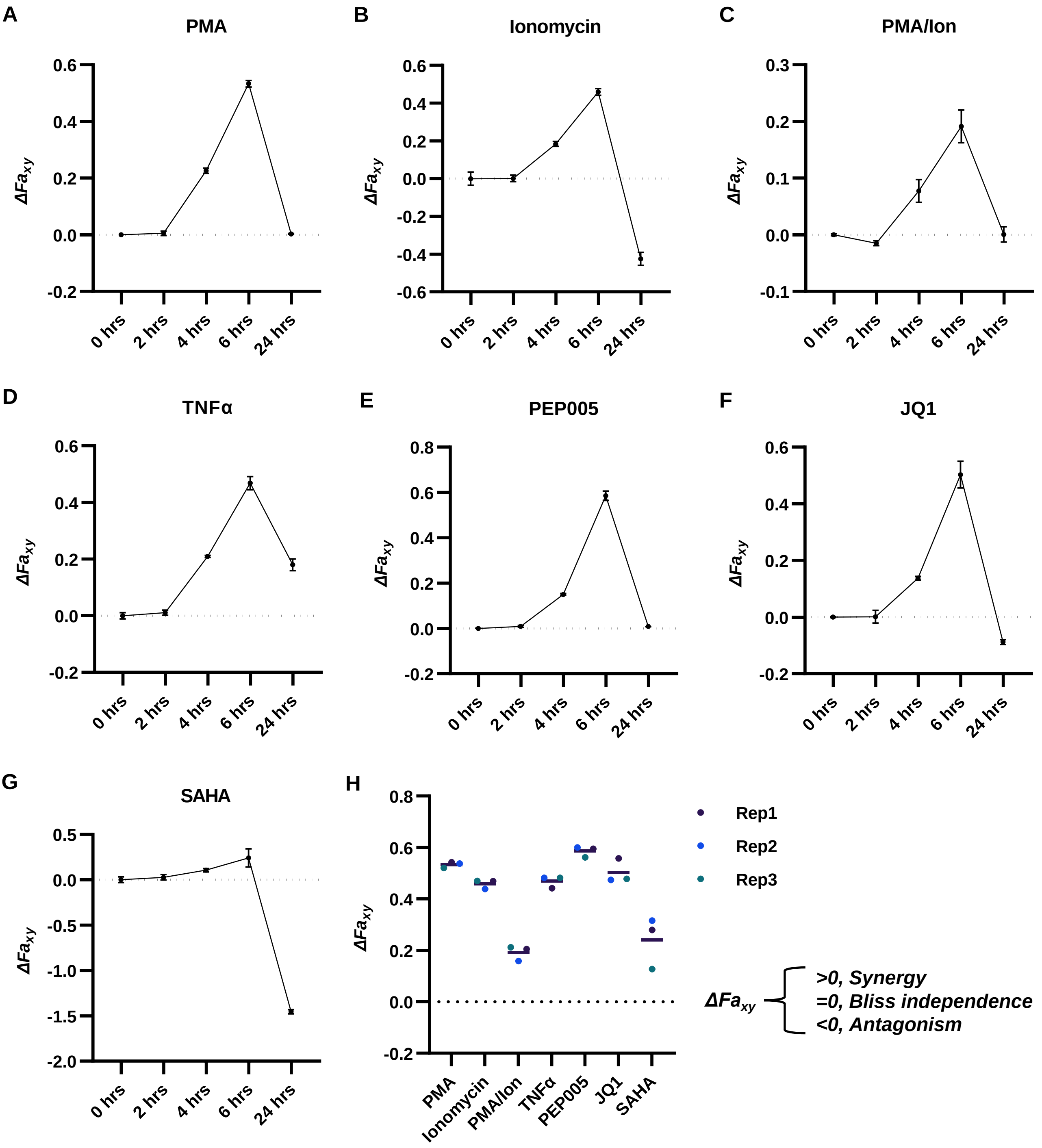
A
PMA
B
Ionomycin
C
PMA/Ion
D
TNFα
E
PEP005
F
JQ1
G
SAHA
H
ΔFa$_{xy}$
0 hrs
2 hrs
4 hrs
6 hrs
24 hrs
PMA
Ionomycin
PMA/Ion
TNFα
PEP005
JQ1
SAHA
Rep1
Rep2
Rep3
ΔFa$_{xy}$
>0, Synergy
=0, Bliss independence
<0, Antagonism

Figure S3

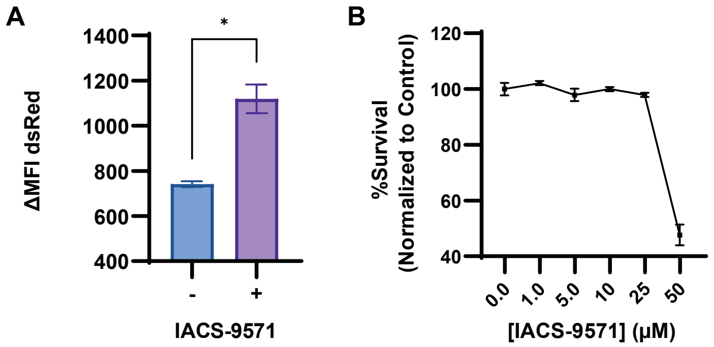
A
*
1400
1200
1000
800
600
400
ΔMFI dsRed
-
+
IACS-9571
B
120
100
80
60
40
%Survival
(Normalized to Control)
0.0
1.0
5.0
10
25
50
[IACS-9571] (μM)

Supplementary Figure 4

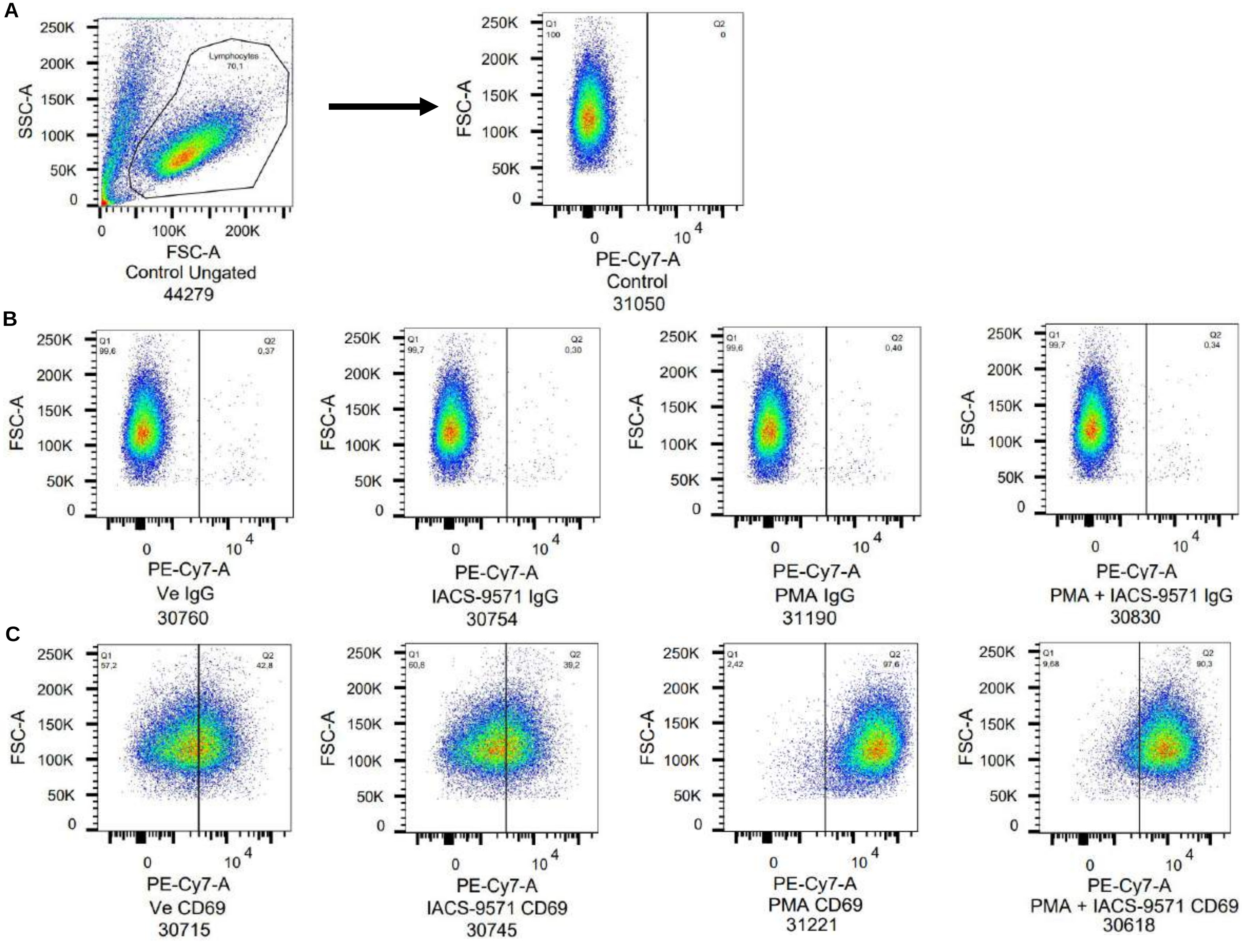

Figure S5

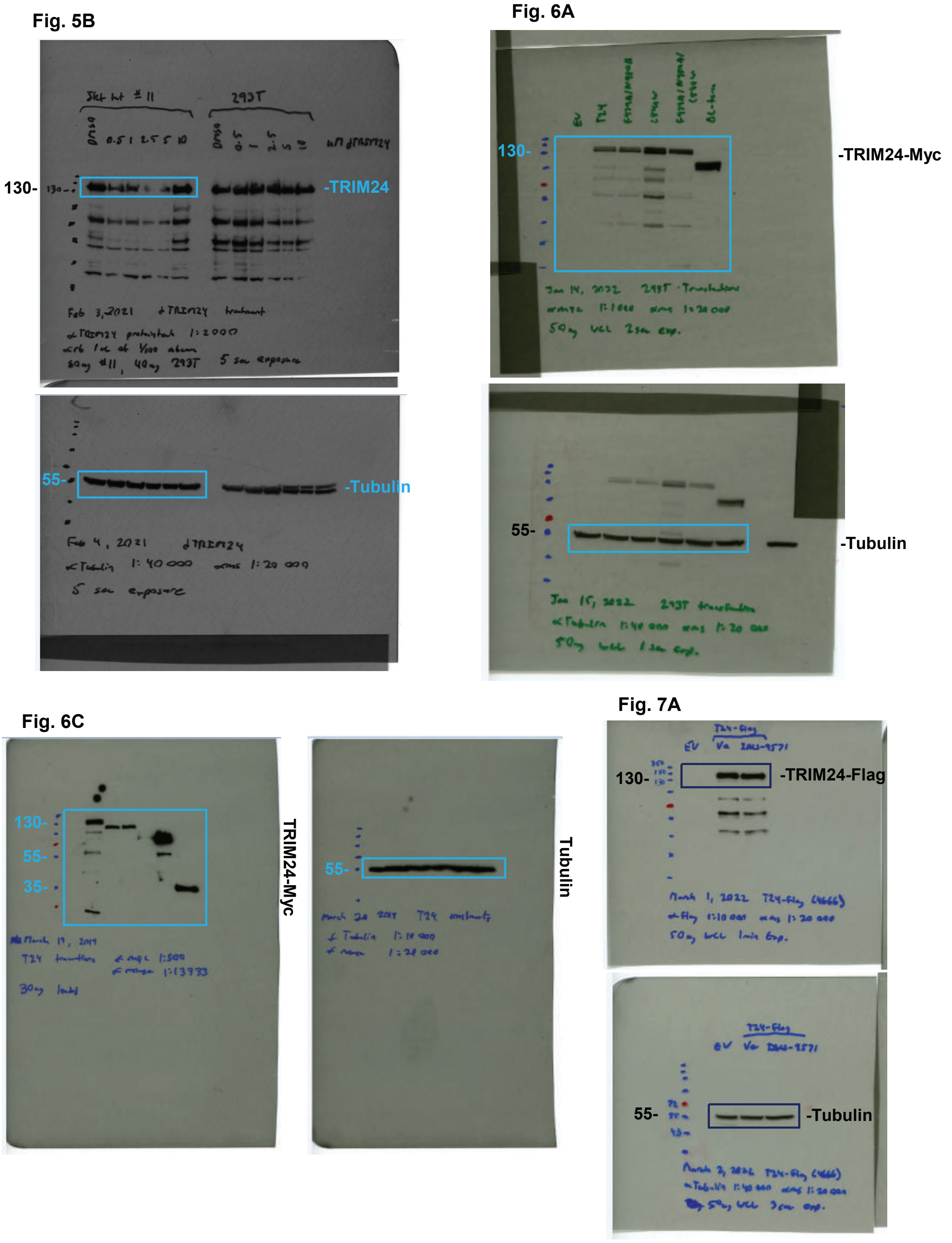
Fig. 5B
130-
-TRIM24
55-
-Tubulin
Fig. 6A
130-
-TRIM24-Myc
55-
-Tubulin
Fig. 6C
130-
55-
35-
TRIM24-Myc
55-
Tubulin
Fig. 7A
130-
-TRIM24-Flag
55-
-Tubulin
